# A global map of saltmarshes

**DOI:** 10.3897/BDJ.5.e11764

**Published:** 2017-03-21

**Authors:** Chris J Mcowen, Lauren V Weatherdon, Jan-Willem Van Bochove, Emma Sullivan, Simon Blyth, Christoph Zockler, Damon Stanwell-Smith, Naomi Kingston, Corinne S Martin, Mark Spalding, Steven Fletcher

**Affiliations:** 1 UN Environment World Conservation Monitoring Centre (UNEP-WCMC), Cambridge, United Kingdom; 2 The Biodiversity Consultancy, Cambridge, United Kingdom; 3 NIRAS, Cambridge, United Kingdom; 4 The Nature Conservancy, Cambridge, United Kingdom; 5 Marine Institute, Plymouth University, Plymouth, United Kingdom

**Keywords:** saltmarsh, global, habitat, coastal, blue carbon, geospatial

## Abstract

**Background:**

Saltmarshes are extremely valuable but often overlooked ecosystems, contributing to livelihoods locally and globally through the associated ecosystem services they provide, including fish production, carbon storage and coastal protection. Despite their importance, knowledge of the current spatial distribution (occurrence and extent) of saltmarshes is incomplete. In light of increasing anthropogenic and environmental pressures on coastal ecosystems, global data on the occurrence and extent of saltmarshes are needed to draw attention to these critical ecosystems and to the benefits they generate for people. Such data can support resource management, strengthen decision-making and facilitate tracking of progress towards global conservation targets set by multilateral environmental agreements, such as the Aichi Biodiversity Targets of the United Nations' (UN's) Strategic Plan for Biodiversity 2011-2020, the Sustainable Development Goals of the UN's 2030 Agenda for Sustainable Development and the Ramsar Convention.

**New information:**

Here, we present the most complete dataset on saltmarsh occurrence and extent at the global scale. This dataset collates 350,985 individual occurrences of saltmarshes and presents the first global estimate of their known extent.

The dataset captures locational and contextual data for saltmarsh in 99 countries worldwide. A total of 5,495,089 hectares of mapped saltmarsh across 43 countries and territories are represented in a Geographic Information Systems polygon shapefile. This estimate is at the relatively low end of previous estimates (2.2-40 Mha), however, we took the conservative approach in the mapping exercise and there are notable areas in Canada, Northern Russia, South America and Africa where saltmarshes are known to occur that require additional spatial data. Nevertheless, the most extensive saltmarsh worldwide are found outside the tropics, notably including the low-lying, ice-free coasts, bays and estuaries of the North Atlantic which are well represented in our global polygon dataset. Therefore, despite the gaps, we believe that, while incomplete, our global polygon data cover many of the important areas in Europe, the USA and Australia.

## Introduction

Saltmarshes are highly productive ecosystems rich in biodiversity, consisting of a variety of plant species that provide important habitats for a wealth of fauna. In addition, they contribute to livelihoods locally and globally through the associated ecosystem services they provide. In particular, they are recognised as important carbon sinks that protect coastal areas from natural disasters, such as erosion and storm surges. Tidal saltmarsh communities (hereafter saltmarshes) comprise the upper, vegetated portion of intertidal mudflats, lying approximately between mean high water neap tides and mean high water spring tides. Saltmarshes occur worldwide, particularly in middle to high latitudes, and are usually restricted to comparatively sheltered locations. The vegetation of a saltmarsh consists of halophytic (salt-tolerant) herbs, grasses and low shrubs adapted to regular or occasional immersion by the tides. Within the saltmarsh community, there are often clear patterns of zonation, typically linked to inundation and salinity. Saltmarshes can be highly dynamic - prone to erosion, but also rapidly colonizing new sediments. Their distribution is not only influenced by, but also influences local physical processes and geomorphology including topography and creek patterns (Adam 1990). The species composition and type of saltmarsh varies from site to site and regionally (Fischer et al. 2000).

Saltmarshes are unique ecosystems, situated at the interface between terrestrial and marine ecosystems, and often further connecting saline and freshwater ecosystems. They provide critical habitat for numerous aquatic and terrestrial organisms, which use them as a refuge from predators and as a location to feed, shelter and spawn (Deegan et al. 2000). Furthermore, they act as high tide refuges for birds feeding on adjacent mudflats, as breeding sites for waders, gulls and terns and as a source of food for passerine and migratory birds (Greenberg et al. 2014). In addition, these wetlands provide valuable ecosystem services (Gedan et al. 2009) that are of significant commercial and recreational value. For example, saltmarshes increase the abundance of fish invertebrate stocks of importance ofr both commercial and recreational fishing; high diversity of birds found within marshes make them a popular location for tourists and bird watchers; they protect shorelines from erosion by buffering wave action and they protect water quality by filtering runoff and metabolising excess nutrients (Barbier et al. 2011).

Despite their importance, saltmarsh areas are declining around the world, having lost between 25% and 50% of their global historical coverage (Crooks et al. 2011, Duarte et al. 2008). Vast areas have been lost through conversion to agriculture, urban and industrial land, while remaining saltmarshes face many other current and potential threats (Gedan et al. 2009): land claim for industry, port facilities, transport infrastructure and waste disposal is still comparatively common; invasive species such as Spartina which threatens mudflat fauna and birds but also saltmarsh vegetation itself; many saltmarshes are being 'squeezed' between an eroding seaward edge and fixed flood defence walls and grazing has a marked effect on the structure and composition of saltmarsh vegetation by reducing the height of the vegetation and the diversity of plant and invertebrate species A range of other human activities can be locally damaging, including waste tipping, pollution and turf cutting (Neubauer 2009).

Due to the continued pressures placed upon saltmarshes, it is essential to establish a mapped baseline in order to assess change and enhance management efforts. Recent best estimates of the global area of saltmarsh range from 2.2 to 40 Mha Pendleton et al. 2012). One reason for the relatively large range in estimated area is that whist saltmarshes have been mapped locally, and even nationally in many places, there has never been a systematic attempt to map the distribution and extent of saltmarshes globally. This has prevented any accurate accounting of overall declines in this critical habitat, or indeed of geographic patterns in such declines. This is in stark contrast to other coastal systems such as coral reefs (Spalding et al. 2001), seagrasses (Green and Short 2003), and mangrove forests Giri et al. 2010, Spalding et al. 2010, Spalding et al. 1997).

Synthesis of existing saltmarsh distribution data into a global map began in 2007, but was never formally published. Using the groundwork established previously, the same general approach of constructing a mosaic map from multiple sources was resumed in 2013, and is presented here as the first detailed global map of saltmarshes.

## General description

### Purpose

This composite dataset was developed to provide a baseline inventory of the extent of our knowledge regarding the global distribution of saltmarshes.

### Additional information

This dataset represents a first step toward tracking the status and trends of saltmarshes globally, outlining our current state of knowledge and revealing locations where monitoring should be established or strengthened. Ongoing monitoring is essential to support progress toward the targets set by multilateral environmental agreements, such as the Aichi Biodiversity Targets of the United Nations' (UN's) Strategic Plan for Biodiversity 2011-2020, the Sustainable Development Goals of the UN's 2030 Agenda for Sustainable Development and the Ramsar Convention, with wetlands contributing substantially to achieving associated targets (see Ramsar Convention Secretariat (2016)). Datasets such as these can also help to inform global monitoring initiatives such as the Group on Earth Observations Wetlands (GEO-Wetlands) Initiative and the associated Global Wetlands Observing System (GWOS), which will draw from ongoing projects such as the Satellite-based Wetland Observation Service (SWOS) and GlobWetland-Africa. The dataset has direct application to indicator development through use in the Wetland Extent Trends (WET) Index (Dixon et al. 2016), for instance.

Knowledge of the extent of saltmarshes is important for calculating potential carbon storage in a manner akin to that done for mangroves (Hutchison et al. 2013) and seagrasses (Fourqurean et al. 2012), which can support decision-makers in implementing climate change mitigation measures and spatial planners in conducting area-based planning. This dataset and other similar datatsets outlining habitat distribution have also been used to support high-level screening by industry (Martin et al. 2015), offering a preliminary overview of areas of importance to biodiversity that can then be verified through *in situ* surveys.

This dataset will be updated as new data become available. We invite those with knowledge of saltmarsh occurrence and extent, or geospatial data that would improve the quality or extent of our knowledge - particularly with regards to change over time - to contact us.

The data are available as a compressed folder containing the following resources:

1. Polygon shapefile containing known distribution of saltmarshes, with contextual information (for spatial analysis; Table 1);
2. Point shapefile containing locations of saltmarsh, linked to an Access database with information on species present at each location (for reference purposes Table 2);
3. Table of data providers;
4. Detailed metadata sheet; and
5. Excel spreadsheet outlining definitions of saltmarshes by country and language.

## Sampling methods

### Sampling description

This dataset is composed of data derived from peer-reviewed articles and grey literature, including reports and databases created by governmental and non-governmental organisations, universities, institutes and researchers globally.

Data in the original source materials were collected using remote sensing and field-based survey methods, with data quality ranging from high-resolution maps to low-resolution representations. Inital work in compiling this dataset was led and funded by The Nature Conservancy with support from United Nations Environment World Conservation Monitoring Centre (UNEP-WCMC), with the second phase led and funded by UNEP-WCMC with contributions from Conservation International. As part of this process, detailed descriptions of the datasets were obtained and recorded within the associated metadata. Where available, these included the time frame of data collection, source(s), resolution and methods of processing.

### Quality control

Data contributors were asked to submit metadata with each dataset to provide the contextual information important for informing analyses. Saltmarshes were identified in accordance with definitions provided by data sources, where available, or were determined using expert opinion (available in the supplementary material).

The precautionary principle was applied to ensure consistency across designations e.g if we were uncertain about the classification of the habitatit was not included. Please refer to the supplementary material and associated metadata for more information regarding each data source presented. Where overlaps occurred, newer or ground-truthed data were prioritised. If equivalent in quality, overlapping and adjacent datasets were incorporated into the layer and dissolved to calculate total area.

Although data outlining mangrove extent are not included in the dataset, these habitats overlap with saltmarshes in some subtropical and tropical regions, with mangrove trees growing amongst saltmarsh, or with clear zones across the intertidal belt dominated by marsh plants or by mangroves. We therefore expect there to be some overlap between maps of these two habitats, although we have not yet "cleaned" these or assessed the level of overlap. The dataset contains a broad range of saltmarsh habitats, for example, the grass-dominated systems which are prevelant in the Eastern United states; the more shrub dominated systems of northern Europe and tundra based systems in Russia.

This map is not intended to reflect a comprehensive assessment of saltmarsh presence and absence, but rather an overview of the extent of our knowledge and data availability with regards to the locations of these habitats globally. While some datasets within the compilation document the time frame of data collection, the dataset as a whole cannot be used for temporal analyses of change due to an incomplete systematic survey of saltmarsh extent globally over time. Other challenges encountered when collating spatial habitat data globally include differing spatial resolutions (1:10,000 to 1:4,000,000); data collection methodologies (e.g. field surveys, remote sensing); licenses and use restrictions; spatial formats and data quality. Not all data records were validated through *in situ* surveys (denoted within the dataset), and some records were only available as points rather than polygons. While lacking estimates of area, these points provide important information on the location of these saltmarshes and the halophytic species known to occur in the region (through the accompanying Access database).

## Geographic coverage

### Description

The dataset captures locational and contextual data for saltmarsh in 99 countries worldwide. The scale of the data varies from 1:10,000 to 1:4,000,000, with most falling within the 1:10,000 to 1:100,000 range.

## Taxonomic coverage

### Description

Saltmarshes were identified in accordance with definitions provided by data sources, where available, or were determined using expert opinion (available in the supplementary material).

## Temporal coverage

**Data range:** 1973-1-01 – 2015-1-01.

### Notes

The time frames for data collection range from 1973 to 2015, with most occuring after 2005

## Usage rights

### Use license

Other

### IP rights notes

The dataset is curated and distributed (http://data.unep-wcmc.org/datasets/43) by the UN Environment World Conservation Monitoring Centre. The dataset is to be used in accordance with the terms and conditions of Attribution-NonCommercial 3.0 Unported (CC BY-NC 3.0) (https://creativecommons.org/licenses/by-nc/3.0/)

## Data resources

### Data package title

A global map of saltmarshes​

### Number of data sets

1

### Data set 1.

#### Data set name

Global saltmarsh distribution

#### Number of columns

1

#### Download URL

http://data.unep-wcmc.org

#### 

**Data set 1.** 

Column label	Column description
A global map of saltmarshes	A global map of saltmarshes

## Figures and Tables

**Table 1.** Recorded salt marsh extent by region (in hectares), number of polygons, and time frame and high-level methodology of data collection, as derived from the “Global Distribution of Saltmarsh” polygon dataset.

	**Region**	Area (Ha)*	**Number**	**Start Date**	**End Date**	**Methodology**
**North and Central America**	USA (Mainland, Hawa'ii)	1,723,410	123,697	01/01/1977	31/12/2012	Remote sensing, field survey
	Mexico	272,527	437	N/A	N/A	Remote sensing
	USA (Alaska)	161,483	8,949	01/01/1977	31/12/2012	Remote sensing, field survey
	Canada	111,274	10,502	01/01/1995	31/12/2002	Field survey
**South America**	Argentina	118,870	3,086	01/03/2000	28/02/2003	Remote sensing, field survey
	Brazil, Uruguay, Chile, Peru	37,858	385	01/03/2000	31/12/2011	Remote sensing, field survey
**Africa and the Middle East**	South Africa	6,147	1,561	01/01/2008	31/12/2008	Remote sensing
	Madagascar	5,810	4	01/01/2011	31/12/2011	Remote sensing, field survey
	United Arab Emirates	4,797	174	07/10/2011	20/05/2014	Remote sensing
**Europe**	Mainland Europe**	356,947	31,830	01/01/1999	13/02/2015	Remote sensing, field survey, ground-truthed
	Great Britain	81,842	117,052	01/01/2006	31/12/2009	Remote sensing, ground-truthed
	Ireland (Republic of)	9,889	13,127	01/01/2006	31/12/2008	Remote sensing, field survey
	Iceland	2,617	32	01/01/2006	31/12/2006	Remote sensing
**Russian Federation**		700,719	50	01/07/1973	31/07/2011	Field survey
**China**		549,506	21,958	01/01/1999	31/12/2008	Remote sensing
**Oceania**	Australia	1,325,854	14,145	01/01/2001	31/12/2001	Remote sensing, ground-truthed
	New Zealand	19,650	2,482	01/01/2007	31/12/2008	Remote sensing
**Small Island Developing States**	Puerto Rico and the U.S. Virgin Islands	5,879	1,498	01/01/1977	31/12/2012	Remote sensing, field survey
	Guam and the Commonwealth of Northern Marianas	8.2	13	01/01/1977	31/12/2012	Remote sensing, field survey
	American Samoa	0.1	3	15/12/2003	15/12/2003	Remote sensing, field survey
	**TOTAL**	**5,495,089**	**350,985**			
* Area calculated after dissolving to remove overlapping polygons. ** Coverage in mainland Europe includes 20 countries: Albania, Belgium, Bulgaria, Croatia, Cyprus, Denmark, Estonia, Finland, France, Germany, Italy, Latvia, Montenegro, Netherlands, Portugal, Romania, Slovenia, Spain, Sweden and Turkey.						

**Table 2.** The number of saltmarsh point locations per country in the “Global Distribution of Saltmarsh” point dataset.

	**Country**	**Number of point records**
**North and Central America**	Antigua and Barbuda	1
	Canada	25
	Costa Rica	2
	Cuba	3
	Greenland	5
	Mexico	15
	Nicaragua	1
	Panama	1
	United States	1
**South America**	Argentina	20
	Brazil	6
	Chile	11
	Ecuador	4
	Peru	1
	Uruguay	7
	Venezuela	4
**Africa and the Middle East**	Algeria	2
	Angola	5
	Djibouti	1
	Egypt	8
	Gambia	2
	Ghana	2
	Iran (Islamic Republic of)	5
	Iraq	1
	Kenya	2
	Kuwait	5
	Mauritania	3
	Morocco	3
	Namibia	2
	Oman	1
	Pakistan	2
	Qatar	2
	Saudi Arabia	14
	Somalia	1
	South Africa	12
	Sudan	1
	Tanzania, United Republic of	1
	Tunisia	2
	United Arab Emirates	8
	Western Sahara	1
**Europe**	Albania	8
	Azerbaijan	1
	Bulgaria	1
	Croatia	2
	Estonia	47
	Georgia	2
	Greece	83
	Iceland	3
	Latvia	8
	Lithuania	1
	Norway	7
	Poland	9
	Slovenia	1
	Spain	2
	Sweden	5
	Ukraine	4
**Russian Federation**	Russia	29
**Oceania**	American Samoa	2
	Cook Islands	1
	Fiji	1
	New Caledonia	1
	Tonga	2
	Vanuatu	2
**Asia**	Bahrain	4
	Cambodia	1
	India	6
	Japan	9
	Korea, DPR	1
	Korea, Republic of	3
	Philippines	2
	Sri Lanka	10
	Taiwan	1
	Viet Nam	10

## References

[B3528471] Adam Paul (1990). The saltmarsh biota. Saltmarsh ecology.

[B3528485] Barbier Edward B., Hacker Sally D., Kennedy Chris, Koch Evamaria W., Stier Adrian C., Silliman Brian R. (2011). The value of estuarine and coastal ecosystem services. Ecological Monographs.

[B3528519] Crooks Stephen, Herr Dorothee, Tamelander Jerker, Laffoley Dan, Vandever Justin (2011). Mitigating climate change through restoration and management of coastal wetlands and near-shore marine ecosystems : challenges and opportunities.. Environment department papers ; no. 121. Marine ecosystem series..

[B3528535] Deegan Linda A., Hughes Jeffrey E., Rountree Rodney A., Weinstein MP, Kreeger DA (2000). Salt Marsh Ecosystem Support of Marine Transient Species. Concepts and Controversies in Tidal Marsh Ecology.

[B3528551] Dixon M. J.R., Loh J., Davidson N. C., Beltrame C., Freeman R., Walpole M. (2016). Tracking global change in ecosystem area: The Wetland Extent Trends index. Biological Conservation.

[B3528563] Duarte Carlos M., Dennison William C., Orth Robert J. W., Carruthers Tim J. B. (2008). The Charisma of Coastal Ecosystems: Addressing the Imbalance. Estuaries and Coasts.

[B3528573] Fischer Janet M., Reed-Andersen Tara, Klug Jennifer L., Chalmers Alice G. (2000). Spatial Pattern of Localized Disturbance along a Southeastern Salt Marsh Tidal Creek. Estuaries.

[B3528583] Fourqurean James W., Duarte Carlos M., Kennedy Hilary, Marbà Núria, Holmer Marianne, Mateo Miguel Angel, Apostolaki Eugenia T., Kendrick Gary A., Krause-Jensen Dorte, McGlathery Karen J., Serrano Oscar (2012). Seagrass ecosystems as a globally significant carbon stock. Nature Geoscience.

[B3528600] Gedan K. Bromberg, Silliman B. R., Bertness M. D. (2009). Centuries of Human-Driven Change in Salt Marsh Ecosystems. Annual Review of Marine Science.

[B3528644] Giri C., Ochieng E., Tieszen L. L., Zhu Z., Singh A., Loveland T., Masek J., Duke N. (2010). Status and distribution of mangrove forests of the world using earth observation satellite data. Global Ecology and Biogeography.

[B3528658] Greenberg Russell, Cardoni Augusto, Ens Bruno J., Gan Xiaojing, Isacch Juan Pablo, Koffijberg Kees, Loyn Richard (2014). The distribution and conservation of birds of coastal salt marshes. Coastal Conservation.

[B3528685] Green EP, Short FT (2003). World Atlas of Seagrasses.

[B3528694] Hutchison James, Manica Andrea, Swetnam Ruth, Balmford Andrew, Spalding Mark (2013). Predicting Global Patterns in Mangrove Forest Biomass. Conservation Letters.

[B3528705] Martin C. S., Tolley M. J., Farmer E., Mcowen C. J., Geffert J. L., Scharlemann J. P.W., Thomas H. L., Bochove J. H. van, Stanwell-Smith D., Hutton J. M., Lascelles B., Pilgrim J. D., Ekstrom J. M.M., Tittensor D. P. (2015). A global map to aid the identification and screening of critical habitat for marine industries. Marine Policy.

[B3528755] Neubauer Scott C. (2009). Silliman, B. R., E. D. Grosholz, and M. D. Bertness (ed.) Human Impacts on Salt Marshes: A Global Perspective. Wetlands.

[B3528725] Pendleton Linwood, Donato Daniel C., Murray Brian C., Crooks Stephen, Jenkins W. Aaron, Sifleet Samantha, Craft Christopher, Fourqurean James W., Kauffman J. Boone, Marbà Núria, Megonigal Patrick, Pidgeon Emily, Herr Dorothee, Gordon David, Baldera Alexis (2012). Estimating Global “Blue Carbon” Emissions from Conversion and Degradation of Vegetated Coastal Ecosystems. PLoS ONE.

[B3528746] Secretariat Ramsar Convention (2016). The Fourth Ramsar Strategic Plan 2016-2024. Ramsar handbooks for the wise use of wetlands..

[B3528774] Spalding MD, Blasco F, Field CD (1997). World Mangrove Atlas.

[B3528765] Spalding M, Kainuma M, Collins L (2010). World Atlas of Mangroves.

[B3528793] Spalding MD, Rabillious C, Green EP (2001). World Atlas of Coral Reefs.

